# Potentially inappropriate medications in elderly ambulatory and institutionalized patients: an observational study

**DOI:** 10.1186/s40360-016-0081-x

**Published:** 2016-08-21

**Authors:** Daniela Petruta Primejdie, Marius Traian Bojita, Adina Popa

**Affiliations:** 1 Department of Clinical Pharmacy, “Iuliu Hatieganu” University of Medicine and Pharmacy, Faculty of Pharmacy, 12 Ion Creanga St, 400010 Cluj-Napoca, Romania; 2 Department of Pharmaceutical Analysis, “Iuliu Hatieganu” University of Medicine and Pharmacy, Faculty of Pharmacy, 6 Louis Pasteur St, 400349 Cluj-Napoca, Romania

**Keywords:** Inappropriate prescribing, Drug -related problems, Ambulatory elderly, Institutionalized elderly, STOPP/START criteria, PRISCUS list

## Abstract

**Background:**

The elderly are frequently exposed to drug related problems causing hospitalizations and increased costs of care. Information about Romanian prescribing practices among the elderly and potential medication associated- risks is lacking. The objective of this study was to identify and compare the most frequent potentially inappropriate medications (PIM) recommended to ambulatory and institutionalized Romanian elderly, through an observational retrospective design.

**Methods:**

All reimbursed medications prescribed to a sample of ambulatory elderly accessing two community pharmacies and all medications recommended to a group of institutionalized elderly (urban facilities, Romania, same month) were analyzed. The STOPP/START criteria and the PRISCUS list were used for PIM identification and for classification as misprescribed, underprescribed or overprescribed -subtypes.

**Results:**

The analysis involved 345 prescriptions recommended to ambulatory elderly and 91 medical files available for the institutionalized patients. The ambulatory elderly had a mean age of 74.8 years old and were daily exposed to a median number of 3 prescribed medications. The institutionalized elderly were older (mean age 80.77) received 8 medications daily and 69 % of them were functionally dependent. Cardiovascular and neuropsychiatric indications were the most frequent: 64.34 % and 18.55 % of the ambulatory prescriptions, 93.40 % and 41.75 % of the institutionalized patients’ medical files. 159 PIM were identified on 34.49 % of the ambulatory prescriptions. 82.41 % of the institutionalized patients’ medical files contained 140 PIM. The potential underprescribing of cardiovascular therapies was the most frequent PIM category on the ambulatory prescriptions (55.34 % of all PIM), while for the institutionalized patients’ medical files, the misprescribed and overprescribed PIM were those predominantly represented (62.14 % and 27.14 % of all PIM). In both subgroups of data, NSAIDs (56.66 % of ambulatory prescriptions and 35.63 % of institutionalized patients’ data) and benzodiazepines (26.66 % of ambulatory prescriptions and 24.13 % of institutionalized patient’s data) were predominantly misprescribed. Anticholinergics were rarely used (0.62 % of total PIM from ambulatory prescriptions, 2.14 % of total PIM from institutionalized patients’ data).

**Conclusions:**

The PIM identified in both elderly groups suggested potential risks for the occurrence of adverse events specific to the elderly population. Larger studies, both observational and interventional, are needed to ensure a safer therapeutic approach.

## Background

Age-related changes influence the pharmacokinetic pathways and the pharmacodynamic response of therapies delivered to the elderly population, increasing the risk for drug-related problems (DRP) which favor costly and frequent hospitalizations, and augmenting the mortality risk, irrespective of the environment of care [1–4]. The variability of the individual degree of functional independence is another significant aspect influencing therapeutic decisions that need to ensure functional independence and prolong social insertion [5].

The potentially inappropriate medications (PIM) and the corresponding phenomenon of potentially inappropriate prescribing (PIP) refer to the use of medications identified through various studies as factors that expose the elderly patient to safety or efficacy DRP. The literature suggests three subtypes for PIM: misprescribed PIM involving medications with an increased risk for side effects or drug-interactions in the elderly, underprescribed PIM relating to the absence of medications proved to be effective for preventive or curative indications in the elderly, and overprescribed PIM relating to the use of therapies lacking therapeutic benefit [6, 7].

Such PIM can be identified in an individual therapeutic plan, using published explicit criteria consisting of lists of medications to avoid, to use cautiously or to actively recommend to the elderly. Several such validated instruments are available in the literature, having different structures and medications: the Beers criteria (USA), the STOPP/START (Screening Tool of Older Persons’ Prescriptions/Screening Tool to Alert doctors to Right, i.e. appropriate, indicated Treatment) criteria (Ireland), the PRISCUS (Latin for “old and venerable”) list (Germany) etc [8–10]. The potential warning function of such instruments was confirmed through various investigations that intended to substantiate a relationship between PIM use and negative outcomes in the elderly. For example, the use of medications included in the Beers 2012 and STOPP criteria was associated with admittance to the emergency department for 30.3 % of the nursing home residents, followed by Grace et al. in a retrospective cohort study, while Matanović et al. found that 54 % of hospitalizations registered for 454 consecutive ambulatory patients were associated with the use of non-COX-selective NSAIDs, short- and intermediate-acting benzodiazepines, and amiodarone, identified using Beers criteria [11, 12]. Frankenthal et al. successfully used STOPP/START as an instrument to influence misprescribing in a chronic care geriatric facility. Therefore, there was a reduction in the number of prescribed medications, therapy – associated costs and in the average number of falls [13]. Furthermore, some of these instruments were also used as quality measures in several insurance programs, confirming their applicability in large population groups [8]. However, various studies showed that PIM identification with these instruments depends on local availability of the included medications and on local patterns of geriatric practice [14, 15]. Therefore, the simultaneous use of several such instruments was suggested as a more efficient method for PIM detection in elderly populations different from those used as validation group for the original criteria [16].

The local population follows the aging trend identified at an international level while the hospitalization rate among the Romanian elderly has an annual increase of 17.82 % [17]. Romanian elderly generally have access to prescribed medications, nonprescription medication and to various natural products. The prescribed medications can be partially or totally reimbursed by the national system of heath care insurance and the community pharmacies keep an electronic and printed record of the reimbursed dispensed medications. The nonprescription medications and natural products are neither reimbursed nor recorded. Professional activities such as medication review of the elderly pharmacotherapy are not mandatory and are usually conducted in an academic or research- related context. Detailed information about the quality of medications used by the Romanian elderly is lacking, but it is needed as it could suggest potential directions for safety and efficacy optimization. Therefore, the objective of this study was to identify the PIM recommended for two subgroups of Romanian elderly patients, ambulatory and institutionalized, using the STOPP/START criteria and the PRISCUS list.

## Methods

In this descriptive observational study, two collections of data were analyzed: all reimbursed medications prescribed to a sample of ambulatory elderly accessing two community pharmacies and, respectively, all medications recommended to a group of institutionalized elderly (urban facilities, Romania). All patients were over 65 years old at the time of the data collection and both data sets covered the same 30-day interval (March 2013).

A prescription could contain one to several medications indicated for various conditions.

The pharmacies were selected by convenience as they were considered to be representative for the majority of local community pharmacies in terms of accessibility to the elderly population and orientation towards patient's care (one had an independent- pharmacist owner and the other was included in a national chain of community pharmacies). Both offered services and medications to different population groups. For the ambulatory elderly, the analyzed data excluded details involving the use of nonprescription medications and natural products, as their use was not registered in the available data collection. For the institutionalized elderly, the analysis included all recommended medications (irrespective of their reimbursement status), following the indications of the primary-care physician who attended the facility. The medical files of the institutionalized elderly had variable levels of information related to medical history and laboratory investigations.

The analysis used three sets of PIM identification criteria: STOPP/START, the 2008 version (covering instances of misprescribing, overprescribing and underprescribing) and the PRISCUS list (misprescribing and overprescribing) [9, 10]. These sets of criteria are different in terms of need for patient's medical and medications history: the use of the STOPP/START tool frequently requires access to patients’ clinical information, while the PRISCUS list can be applied without such detail. The availability of the medications or drug classes mentioned in these evaluation criteria was considered as adequate for the descriptive purpose of the study: 63 (94 %) of the 65 medications or drug classes included in the STOPP criteria, 22 of the 22 (100 %) medications or drug classes mentioned in the START criteria, 45 of the 83 (54 %) medications or drug classes included in the PRISCUS list.

The study had a retrospective and observational character and formal consent was not required. The data analysis ensured patient’s confidentiality. The project received the approval of the local ethics committee. Two clinical pharmacists collected and analyzed the information available in the two data sets, using specific data collection forms. Descriptive statistics were used (Microsoft® Office Excel 2007, Microsoft Corporation).

## Results

The two pharmacies delivered 345 prescriptions to the elderly ambulatory patients (34.36 % from a total of 1,004 reimbursed prescriptions delivered to all the patients accessing the pharmacies), covering both chronic and acute indications (Table 1). The proportion of prescriptions delivered to the elderly patients differed between the two pharmacies. For one pharmacy, the prescriptions delivered to the elderly ambulatory patients covered 48.64 % of all reimbursed prescriptions (180 of 370 prescriptions), while for the other pharmacy, they represented 26.01 % of all reimbursed prescriptions (165 of 634 prescriptions). Among the 91 institutionalized elderly 58 (64 %) of them had dementia, 63 (69 %) were functionally dependent (severe dementia or bedridden), while 10 elderly (14 %) had severe renal disease (creatinine clearance ≤ 30 ml/min, estimated with the Cockroft Gault equation).

**Table 1:** Patients’ characteristics

	Patients	
	Ambulatory elderly accessing 2 community pharmacies	Institutionalized elderly in one nursing home facility
	Collection of data	
	Prescriptions of reimbursed medications	Medical files with all recommended medications
Items of data included in each collection	345 prescriptions	91 medical files
mean age (±SD) (years old)	74.8 (±6.24)	80.77 (±6.82)
range of individual age	65-92 years old	65-98 years old
women (% of the elderly patients, in the each population group)	211 (61.16 %)	53 (58.24 %)
age groups		
65-75 years old	193 (55.94 %)	21 (23.08 %)
76-85 years old	129 (37.39 %)	48 (52.75 %)
>85 years old	23 (6.66 %)	22 (24.17 %)
number of medications in each collection of data	1111	752
median number of medications/item of data	3	8
Diagnoses mentioned in each collection of data		
total number of diagnoses	748	399
median number of diagnoses/item of data	2	4
Frequency of types of diagnoses in each collection of data		
Type of diagnosis	number of prescriptions with the respective type of diagnosis (% of the total number of prescriptions)	number of medical files with the respective type of diagnosis (% of the total number of medical files)
cardiovascular	222 (64.34 %)	85 (93.40 %)
neuropsychiatric	64 (18.55 %)	38 (41.75 %)
rheumatologic	61 (17.68 %)	33 (36.26 %)
diabetes	58 (16.81 %)	14 (15.38 %)
gastrointestinal	26 (7.53 %)	12 (13.18 %)
respiratory	28 (8.11 %)	5 (5.49 %)
urologic	20 (5.79 %)	10 (10.98 %)
ophthalmologic	16 (4.63 %)	10 (10.98 %)
other	25 (7.24 %)	24 (26.37 %)

Considering the three sets of explicit criteria, 119 prescriptions (34.49 %) presented 159 PIM, with an average (±SD) of 0.46 (±0.73) PIM/ambulatory elderly prescription and a maximum of 4 PIM/ambulatory elderly prescription. 75 medical files (82.41 %) contained 140 PIM, with an average (±SD) of 1.53 (±1.12) PIM/institutionalized elderly medical file and a maximum of 6 PIM/institutionalized elderly medical file. The underprescribed PIM were most frequent among the ambulatory elderly, while the misprescribed subtypes were frequent among those institutionalized (Tables 2 and 3).

**Table 2:** Types of potentially inappropriate medications (PIM)

	Analyzed sample of medications	
	1111 medications prescribed to the ambulatory elderly	752 medications recommended to the institutionalized elderly
	Total number of PIM in each sample of medications	
	159	140
Subtype – PIM		
Misprescribed – PIM (% of total number of PIM)	60 (37.73 %)	87 (62.14 %)
Underprescribed - PIM (% of total number of PIM)	88 (55.34 %)	15 (10.71 %)
Overprescribed - PIM (% of total number of PIM)	11 (6.92 %)	38 (27.14 %)

**Table 3:** Potentially inappropriate medications (PIM) identified in the ambulatory and institutionalized elderly pharmacotherapy

	Medications prescribed on 345 reimbursed prescriptions delivered to ambulatory elderly			Medications recommended on 91 medical files of the institutionalized elderly		
Subtype – PIM *Reference criteria* ^*a*^ *: reason to avoid*	no. of examples of subtype- PIM	% of the respective subtype- PIM category	% of 159 PIM	no. of examples of subtype- PIM	% of the respective subtype- PIM category	% of 140 PIM
*Misprescribed – PIM*	60			87		
NSAIDs as chronic analgesics in osteoarthritis *PRISCUS list –analgesics, anti-inflammatory drugs:* – very high risk of gastrointestinal hemorrhage, ulceration, or perforation, which may be fatal – indometacin: central nervous disturbances – phenylbutazone: blood dyscrasia – etoricoxib: cardiovascular contraindications *STOPP tool - Musculoskeletal system* − NSAID with heart failure − Warfarin and NSAID together	34	56.66	21.38	31	35.63	22.14
benzodiazepines *PRISCUS - sedatives, hypnotic agents: * – risk of falling (muscle-relaxing effect) with risk of hip fracture – prolonged reaction times – psychiatric reactions (can also be paradoxical, e.g., agitation, irritability, hallucinations, psychosis) – cognitive impairment – depression	16	26.66	10.06	21	24.13	15.00
antipsychotics *PRISCUS- neuroleptic drugs:* – anticholinergic and extrapyramidal side effects (tardive dyskinesia) – parkinsonism – hypotonia – sedation – risk of falling – increased mortality in demented patients	3	5.00	1.88	18	20.68	12.85
digoxin for heart failure > 0.125 mg/day *PRISCUS list- Antiarrhythmic drugs:* – elevated glycoside sensitivity (women > men) – risk of intoxication *STOPP tool - Cardiovascular system:* - digoxin at a long-term dose > 125 μg/day with impaired renal function	4	6.66	2.51	13	14.94	9.28
anticholinergic drugs (trihexyphenidyl, doxepine) *PRISCUS list- Anticholinergic drugs:* – anticholinergic side effects (e.g., constipation, dry mouth) – impaired cognitive performance – ECG changes (prolonged QT) *PRISCUS list - Tricyclic antidepressants:* – peripheral anticholinergic side effects (e.g., constipation, dry mouth, orthostatic hypotension, cardiac arrhythmia) – central anticholinergic side effects (drowsiness, inner unrest, confusion, other types of delirium) – cognitive deficit – increased risk of falling *STOPP tool - Central nervous system and psychotropic drugs:* – anticholinergics to treat extrapyramidal side effects of neuroleptic medications (risk of anticholinergic toxicity)	1	1.66	0.62	3	3.44	2.14
Duplicate drug classes (two concurrent NSAIDs, ACE inhibitors, ARBs) *STOPP tool - Duplicate drug classes:* - any duplicate drug class prescription.	2	3.33	1.25	1	1.14	0.71
*Underprescribed - PIM*	88			15		
underprescribing of statins in coronary vascular disease *START tool - Cardiovascular system:* - Statin therapy with a documented history of coronary, cerebral or peripheral vascular disease, where the patient’s functional status remains independent for activities of daily living and life expectancy is greater than 5 years	46	52.27	28.93	1	6.66	0.71
underprescribing of β-blocker with chronic stable angina *START tool - Cardiovascular system:* - Beta-blocker with chronic stable angina.	29	32.95	18.23	0	0	0
underprescribing of acenocoumarol^b^ in the presence of chronic atrial fibrillation *START tool - Cardiovascular system:* - Warfarin in the presence of chronic atrial fibrillation.	8	9.09	5.03	0	0	0
underprescribing of antihypertensive therapy where systolic blood pressure consistently > 160 mmHg *START tool - Cardiovascular system:* - Antihypertensive therapy where systolic blood pressure consistently > 160 mmHg.	not applicable^c^			7	46.66	5.00
underprescribing of ACE inhibitors with chronic heart failure *START tool - Cardiovascular system:* - ACE inhibitor with chronic heart failure	3	3.40	1.88	2	13.33	1.42
underprescribing of statins in diabetes mellitus *START tool - Endocrine system:* - Statin therapy in diabetes mellitus if coexisting major cardiovascular risk factors present	1	1.13	0.62	4	26.66	2.85
underprescribing of ACE inhibitors following acute myocardial infarction *START tool - Cardiovascular system:* - ACE inhibitor following acute myocardial infarction	1	1.13	0.62	1	6.66	0.71
*Overprescribed - PIM *	11			38		
overprescribing of anti-dementia drugs, vasodilators, circulation-promoting agents^d^ *PRISCUS - anti-dementia drugs, vasodilators,* *circulation-promoting agents* – no proof of efficacy, unfavorable risk/benefit profile	11	100.00	6.91	38	100.00	27.14

^a^ref. [9, 10]

^b^acenocoumarol is the local available antivitamin K (warfarin in the START criteria)

^c^the information available on the prescription did not include the blood pressure values

^d^Pentoxifylline, *Ginkgo biloba* standardized extract, nicergoline, vinpocetine

*ARB* Angiotensin II Receptor Blocker, *ACE *Angiotensin Converting Enzyme, *NSAIDs* nonsteroidal anti-inflammatory drugs

Percentages are presented as unrounded numbers

For osteoarthritic pain, ambulatory elderly used various non-selective NSAIDs and coxibs, while the institutionalized elderly were exposed to diclofenac and ketoprofen administered intramuscularly. NSAIDs were co-prescribed with cardiovascular medications on 29 (85.30 % of 34) prescriptions and on 25 (80.64 % of 31) medical files presenting NSAIDs. 26 (76.5 % of 34) prescriptions and 27 (87.1 % of 31) medical files had NSAIDs recommended for more than 10 days/month, without concurrent therapy with a proton pump inhibitor. 24 (70.58 %) of NSAIDs prescriptions covered ≤ 20/30 days of analgesic therapy, in the absence of supplemental analgesic approaches. 21 (23.07 %) of the institutionalized elderly presented a diagnosis associated with a persistent pain syndrome, but lacked analgesic therapy. Acetaminophen was not recommended in the nursing home.

Typical and atypical antipsychotics were regularly used in the nursing home. 17 elderly with dementia (29.31 % of all 58 having a dementia diagnosis) received such therapy. Short to long-acting benzodiazepines were recommended for daily use in both environments of care. Vasodilators and circulation-promoting agents (pentoxifylline, *Ginkgo biloba* standardized extract, nicergoline, vinpocetine) were used by 26 (44.82 %) of the 58 institutionalized elderly with dementia and by 17 (26.98 %) of those 63 functionally dependent.

The items from the STOPP/START criteria requiring access to clinical information (medical history, medication history, degree of severity, intolerances or contra-indications etc.) were not applicable for the prescription analysis or for the majority of the included medical files, as the relevant clinical details were not available. For the ambulatory elderly, the STOPP/START criteria referring to the use of aspirin as an antiplatelet agent, of dipyridamole, loperamide or of calcium and vitamin D were not applied, as they refer to over-the-counter medications, not registered in the available database. Several medications from the PRISCUS list, although locally available, were not prescribed: ergotamine and its derivatives, muscle relaxants, baclofen, tetrazepam, clonidine, alpha-blockers, methyldopa, fluoxetine, ticlopidine, prasugrel, nitrofurantoin, sotalol, pethidine, phenobarbital.

## Discussion

This study identified several categories of PIM prescribed to two samples of elderly with different functional capacities and medical complexities, through the use of specific tools validated to this end: the STOPP/START criteria and the PRISCUS list. We found that 34.49 % of the prescriptions delivered to the ambulatory elderly and 82.41 % of the institutionalized patients were exposed to PIM. We decided to analyze these two elderly populations as they represent an appropriate environment for a more active pharmacist’s involvement in the complex process of geriatric care optimization. Although such pharmaceutical care activities are encouraged, their local necessity and benefit, in terms of safety and efficacy of elderly care, are to be demonstrated. Therefore, although our analysis covered a short period of time, it represents a first necessary step in a pilot project aimed at creating a screening instrument for PIM, specific to local prescribing practices and adapted for use by the local community pharmacists. Considering their potential clinical significance, our results could also initiate a pharmacist- prescriber dialogue aiming for a more geriatric- oriented process of care, in terms of both efficacy and safety. Furthermore, both the frequency and nature of the identified PIM suggest the need for further studies evaluating their likely impact on hospitalization rate or care- associated costs.

This is also the first study using the STOPP/START criteria and the PRISCUS list in conjunction, as explicit criteria for PIM identification in two samples of elderly patients cared for in two different environments. As suggested by previous similar research, the methodology used in this observational study tried to overcome the inherent limitations of explicit criteria, through the simultaneous use of three such European instruments [18]. Although several criteria referring to misprescribing instances, included in the STOPP tool overlapped with those of the PRISCUS list, their simultaneous use was considered as useful for the identification of the particular medication use patterns among the two subgroups. As the clinical information necessary for the application of these two sets of criteria is different (the STOPP criteria frequently refers to the patients’ medical or medication background, while the PRISCUS list does not), this aspect was considered appropriate, considering the fact that the prescriptions delivered to the ambulatory patients lacked the clinical detail, while this was present on the medical files of the institutionalized patients. Therefore, the limited access to the patient’s medical history or to information about the use of nonprescription medications proved to be an important although predictable obstacle in the use of all STOPP/START criteria referring to medications locally available. For example the STOPP criteria referring to the use of aspirin as an antiplatelet agent or the START criteria referring to the underuse of effective antihypertensive agents could not be used in the analysis of the ambulatory elderly therapy. This difference in applicability can partially justify the difference in terms of PIM frequency among the 2 sets of data. Moreover, this observation suggests caution in the interpretation of the results as the STOPP/START authors proved that misprescribed PIM are likely to be overestimated using STOPP and underprescribed PIM are likely to be underestimated using START, when the community pharmacist’s analysis lacked patients’ clinical details [19].

This higher exposure to PIM of the institutionalized elderly is a result confirmed in similar comparative studies that found institutionalization as a risk factor for PIP. Haasum et al. found that 30 % of the institutionalized and 12 % of the home-dwelling elderly were exposed to anticholinergic drugs, long-acting benzodiazepines, and concurrent use of 3 or more psychotropics [20]. Shah et al. confirmed this gap in PIM burden among the community and nursing home residents and also found a similar and significant difference in the number of daily medications administered to both populations: 4.9 in the community and 8.4 in care homes (3.22 and 8.26 daily medications, respectively, in our study), suggesting the increased risk for PIP as a consequence of the institutionalized elderly exposure to polypharmacy [21]. Polypharmacy is a prevalent geriatric phenomenon, correlated with increased care-related costs and greater mortality especially among those cognitively impaired [22]. The risk for polypharmacy is increased when medications with debatable benefit are being used, especially among those with limited life expectancy, for whom 40 to 50 % of the recommended medications can be considered as useless or overused [23]. From this point of view, the administration of vasodilators and various circulation-promoting agents (pentoxifylline, *Ginkgo biloba* standardised extract, nicergoline, vinpocetine) recommended as antidementia therapy to both populations, could be considered as potentially overprescribed. They represented 6.91 % of all ambulatory PIM and 27.14 % of all institutionalized elderly PIM, although the evidence of their benefit is reduced and concerns have been expressed regarding the potential for increased hemorrhagic risk, as stated by the PRISCUS criteria [10, 24].

The most frequent PIM categories identified were also different between the two environments of care: the underprescribed (55.34 %) and misprescribed (37.73 %) subtypes prevailed for the ambulatory elderly, while the misprescribed (62.14 %) and overprescribed (27.14 %) subtypes were more frequent in the institutionalized sample. Similarly to our study, Silva et al. used STOPP/START on a sample of institutionalized elderly and found a higher proportion of misprescribed PIM compared to the underprescribed cases: 76.82 % were STOPP criteria and 23.18 % were START criteria in their study [25]. The most prevalent examples were different nevertheless, as in our study the institutionalized elderly were mostly exposed to the high use of NSAIDs and to the underuse of antihypertensive therapy.

The misprescribed and underprescribed PIM identified through the use of STOPP/START criteria are comparable to others available in the literature. For example, a systematic review that included 12 prospective or retrospective observational studies and one randomized clinical trial, which applied full or modified STOPP/START on health records, found a variable frequency of PIM use, influenced by study design, ranging from 21.4 % to 79 %. Some of the identified PIM are similar to those of the present study: recommendations of long-acting benzodiazepines, benzodiazepines with long-acting metabolites, neuroleptics or underuse of statins in patients with documented history of coronary, cerebral or peripheral vascular disease [26]. Several studies that applied the PRISUS list of criteria were also identified. For example, one large-scale German analysis found that 25 % of the elderly received at least one PIM, with amitriptyline, acetyldigoxin, tetrazepam and oxazepam as the most frequent misprescribed PIM [27]. Reich et al. applied the PRISCUS list and the Beers’ 2012 criteria on health care claims data of four health insurers for managed care elderly in Switzerland, and found a 22.5 % estimated prevalence of PIM. PIM use was significantly associated with increasing number of chronic diseases or hospitalizations and again with polypharmacy [28].

The main PIM subtypes identified among the ambulatory elderly were the underprescribing of statins or β-blockers in coronary heart disease (47.16 % of all PIM), recommended to reduce mortality and morbidity even in the elderly population. The clinical relevance of these recommendations suggesting prophylactic approaches with a relative delayed benefit is however limited in the case of the frail elderly, frequently institutionalized, who could have different care needs [29]. Although the data available for the ambulatory elderly did not allow for the assessment of their degree of functionality, these results are similar to others showing a reduced use of prophylactic cardiovascular therapies or a trend of progressive reduction of use of cardiovascular therapies in nursing homes, especially for patients over 80–85 years old [21, 30]. Moreover, we found that 7.69 % of the institutionalized elderly could have benefited from the intensification of the antihypertensive regimen, as indicated by the START criteria, although this approach requires individualization in a population subgroup with a theoretically reduced life-expectancy [31].

The most frequent subtype of misprescribed PIM, identified in both environments of care, was the use of NSAIDs as analgesics in osteoarthritis, representing 56.66 % of total misprescribed -PIM in the ambulatory sample and 35.63 % of total misprescribed -PIM on the medical files, frequently associated with cardiovascular therapies or lacking gastro- protective agents. The amount of clinical information available for the ambulatory elderly could not allow for the assessment of the duration of use or for the presence of renal dysfunction in these patients, but the cardiovascular, gastrointestinal, central nervous system or renal risks remain a serious concern for potential safety- DRP [32]. The results are comparable to those obtained in similar studies that identified a reduced gastrointestinal protection among the elderly exposed to NSAIDs use [33]. The fact that for the institutionalized and generally frailer population, the potential misuse of NSAIDs represented the most frequent instance of PIP suggested the need for the reevaluation of the implemented pain management strategies. Furthermore, the NSAIDs use among other nursing home populations, described in the literature, was significantly lower (1.2 % or 3.8 %), as acetaminophen or opioids represented the preferred analgesic approach [21, 34]. The majority of products containing acetaminophen are available locally without a prescription and therefore they were not reimbursed, so their use could not be monitored in our ambulatory sample. Acetaminophen was not recommended to the institutionalized elderly so questions arise concerning its real use as an analgesic. Furthermore, it is difficult to ascertain the analgesic therapies used by the 71 % of the ambulatory elderly with persistent pain, having less than a 30 days duration recommendation for NSAIDs. Additionally, less than 8 % of the elderly from every sample used weak opioids and adjuvants. Although the underuse of pain medications is not a criterion in the START tool used in this analysis, our findings are in agreement with those identified through a European multicenter analysis, which identified a 48.4 % prevalence of analgesic underuse among institutionalized elderly [35]. Despite the fact that the recently published STOPP/START criteria versions 2 were not available at the time of our data collection, this recent version considers as a START criterion, the use of high-potency opioids in moderate-severe pain and underscores the need for efficient pain management in the elderly [36].

Long-acting benzodiazepines, zolpidem and zopiclone were identified on 4.64 % of the ambulatory elderly prescriptions and on 23.08 % of the institutionalized elderly medical files, for the management of both anxiety or insomnia. These molecules can have negative effects on the cognitive and motor functions, favoring falls and fractures with unproved benefits from long-term use [37, 38]. Bourgeois et al. and de Souto Barreto et al. identified a higher benzodiazepine use (50 % or 53.4 %) among the institutionalized Belgian and French elderly, correlated with the presence of pain syndromes and polypharmacy, but the administration of long half-life molecules or of unadjusted dosages represented potential DRP common to our subgroup [39, 40]. Among the ambulatory 70–89 years old Norwegian population, Neutel et al. found a higher benzodiazepine use that the one identified in our subgroup, with a 12.3 % prevalence of inappropriate use of benzodiazepines as hypnotics or anxiolytics [41]. Almost 20 % of the institutionalized elderly received antipsychotics, with haloperidol as the most frequently prescribed, while their use was infrequent in the ambulatory sample. Our findings are comparable to other similar investigations, as antipsychotic prescribing in Belgian nursing homes varied from 17.6 % to 32.9 % depending on study methodology [42, 43]. Antipsychotic use increases among the demented institutionalized elderly, having as potential indication the control of their neuropsychiatric symptoms or delirium episodes. Their use was associated with extrapyramidal, cardiovascular and cerebrovascular events and with an increased risk of mortality derived especially from the use of typical molecules [10, 44]. Both STOPP and PRISCUS criteria enlist the use of anticholinergic medications as unsafe in the elderly, but our data showed a limited prescription of these molecules (0.62 % of total PIM in the ambulatory elderly and 2.14 % of those institutionalized) by comparison with similar European or North American studies, that identified a 20.7 % and 73.62 % exposure in the institutionalized patients [45, 46]. These findings can be explained by a more reduced availability on the market of the anticholinergic medications presented in the criteria and also by different patterns of medication use in the elderly.

The limits of this study are a consequence of its retrospective nature and of the reduced scale of the data sets used for the analysis, which limit the generalization of these results. Medical history (including cognitive and functionality status) for the ambulatory patients was not available, nor was the information concerning the use of nonprescription medications or natural products. The available clinical detail concerning both collections of data was too limited to allow for the enquiry of several items included in the STOPP/START criteria, which require access to clinical information. The patient’s adherence to the recommended therapies was not known. For both environments of care, the analysis referred to the prescribed medications and not to those actually taken by the elderly patients.

## Conclusions

Our study identified a potentially inappropriate use of NSAIDs as chronic analgesics in osteoarthritis both in ambulatory and institutionalized elderly, a reduced use of prophylactic cardiovascular therapies among the ambulatory elderly and a high overprescribing potential of vasodilators or circulation-promoting agents in the institutionalized sample. The necessity for a more consistent consideration of the risks implied by the aging process became evident. The results of our study encourage their confirmation through more complex investigations or more detailed analysis of the potential impact of the identified PIM on the rate of hospitalization or on care- related costs and could justify future interventional studies intended to optimize the elderly patient's pharmacotherapy.

## Abbreviations

DRP, drug-related problems; NSAIDs, nonsteroidal anti- inflammatory drugs; PIM, potentially inappropriate medications; PRISCUS, latin for “old and venerable”: START, Screening Tool to Alert doctors to Right, i.e. appropriate, indicated Treatment criteria; STOPP, Screening Tool of Older Persons’ prescriptions criteria.
